# Supplementary material for: Apoptotic Activity of New Oxisterigmatocystin Derivatives from the Marine-Derived Fungus Aspergillus nomius NC06
Source: Mar Drugs. 2021 Nov 11;19(11):631. doi: 10.3390/md19110631 (PMC8621937; doi:10.3390/md19110631)
Supplement: Supplementary file 1 [file marinedrugs-19-00631-s001.zip › marinedrugs-1414865-supplementary.pdf]

Apoptotic Activity of New Oxisterigmatocystin Derivatives from the Marine-Derived Fungus Aspergillus n*omius* NC06

Muh. Ade Artasasta [1,2], Yanwirasti Yanwirasti [3], Muhammad Taher [4], Akmal Djamaan [1], Ni Putu Ariantari [5], Ru Angelie Edrada-Ebel [6] and Dian Handayani [1,*]

1 Laboratory of Sumatran Biota/Faculty of Pharmacy, Andalas University, Padang 25163, Indonesia; muh.ade.artasasta.fmipa@um.ac.id (M.A.A.); akmaldjamaan@phar.unand.ac.id (A.D.)
2 Biotechnology Department, Faculty of Mathematics and Natural Sciences, Universitas Negeri Malang (UM), Malang 65145, Indonesia
3 Departement of Biomedical, Faculty of Medicine, Andalas University, Padang 25163, Indonesia; yanwirasti@yahoo.com
4 Faculty of Pharmacy, International Islamic University Malaysia, Kuantan 25200, Malaysia; mtaher@iium.edu.my
5 Department of Pharmacy, Faculty of Mathematics and Natural Sciences, Udayana University, Bali 80361, Indonesia; putu_ariantari@unud.ac.id
6 Strathclyde Institute of Pharmacy and Biomedical Sciences, University of Strathclyde, The John Arbuthnott Building, 161 Cathedral Street, Glasgow G4 0RE, UK; ruangelie.edrada-ebel@strath.ac.uk
* Correspondence: dianhandayani@phar.unand.ac.id; Tel.: +62-751-71682

Supplementary Information

Oxisterigmatocystin J (1)

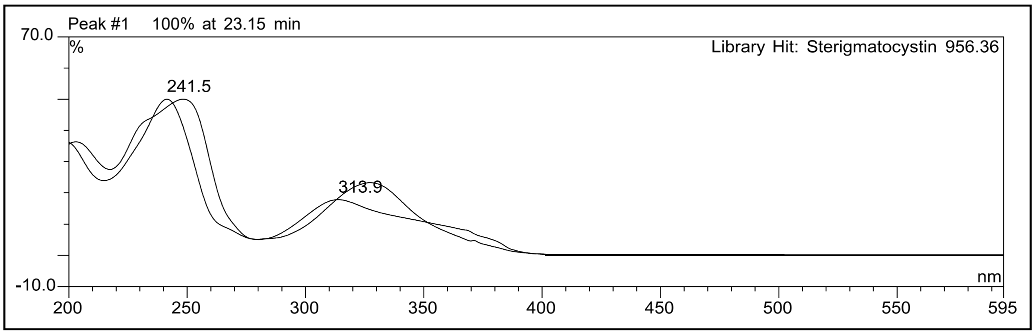

Figure S1. UV spectrum of oxisterigmatocystin J.

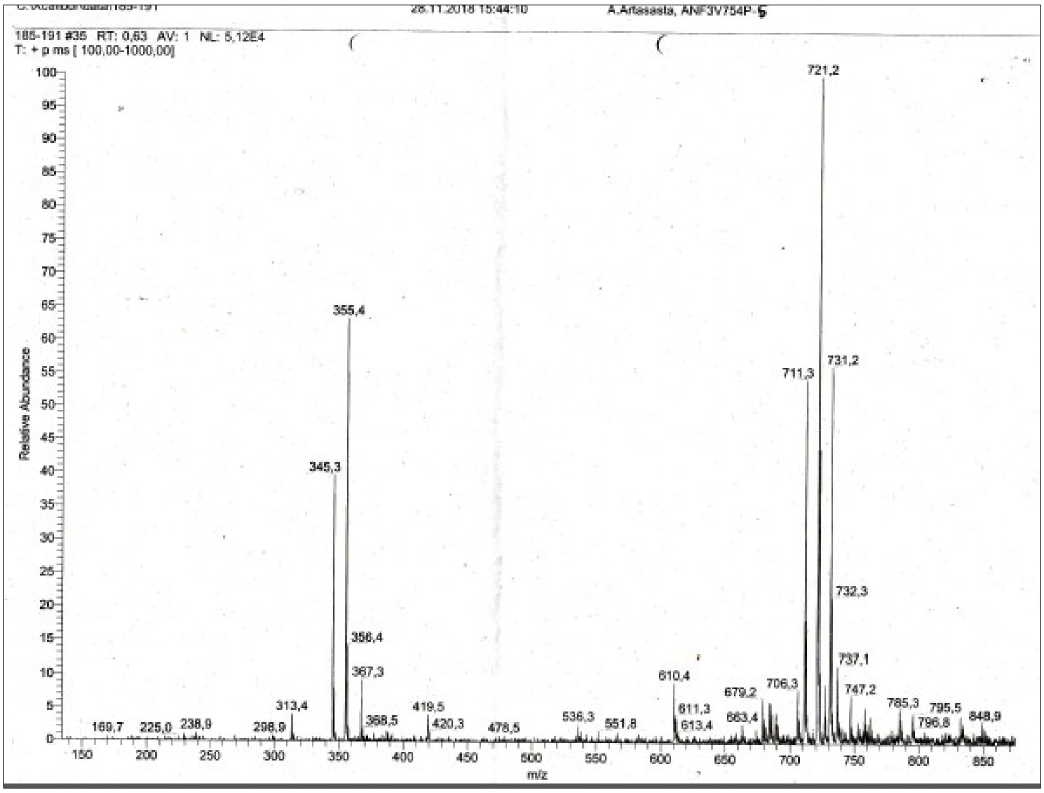

Figure S2. MS spectrum of oxisterigmatocystin J with *m/z* ion of $[M + H]^+$ 355.4.

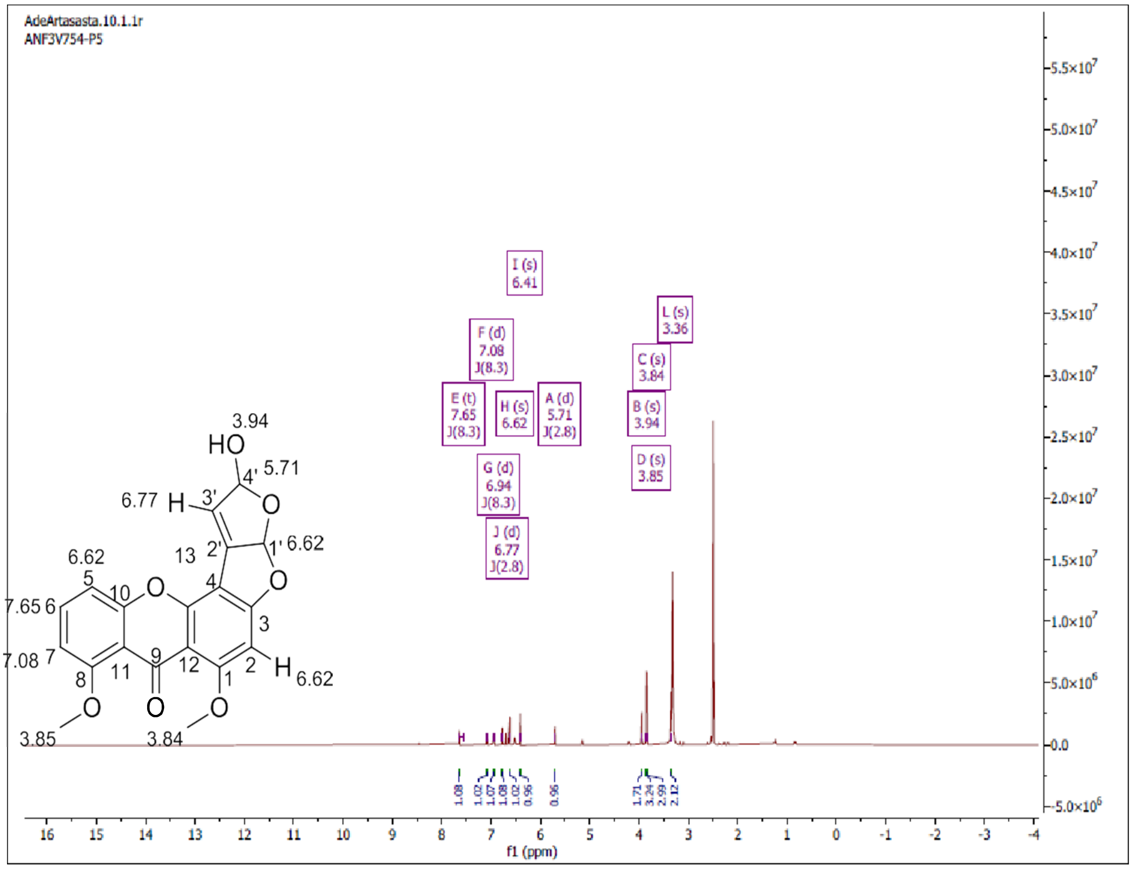

Figure S3. ^{1}H-NMR spectrum of oxisterigmatocystin J.

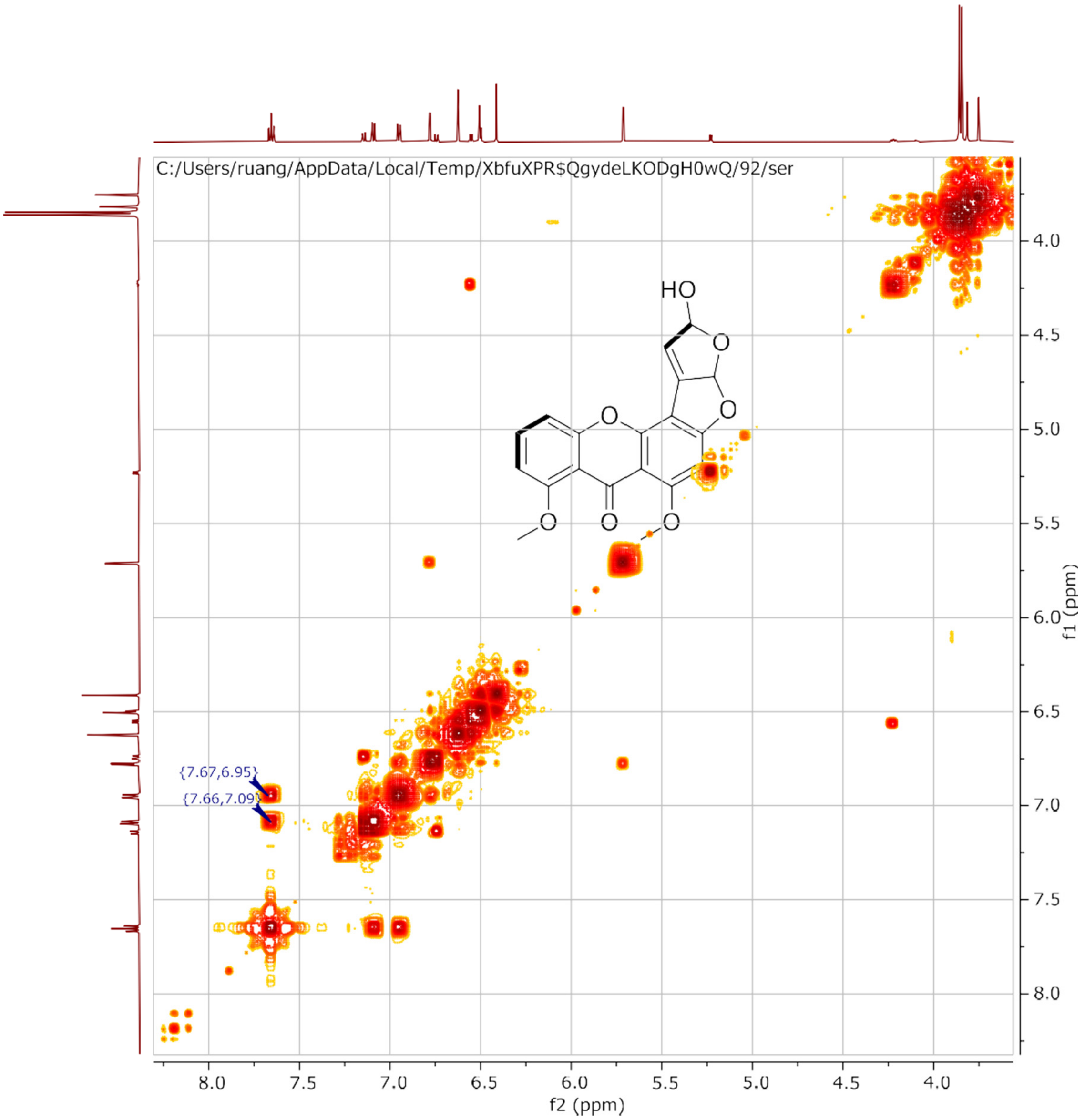

Figure S4. COSY spectrum of oxisterigmatocystin J; proton correlations were shownin black thick bonds.

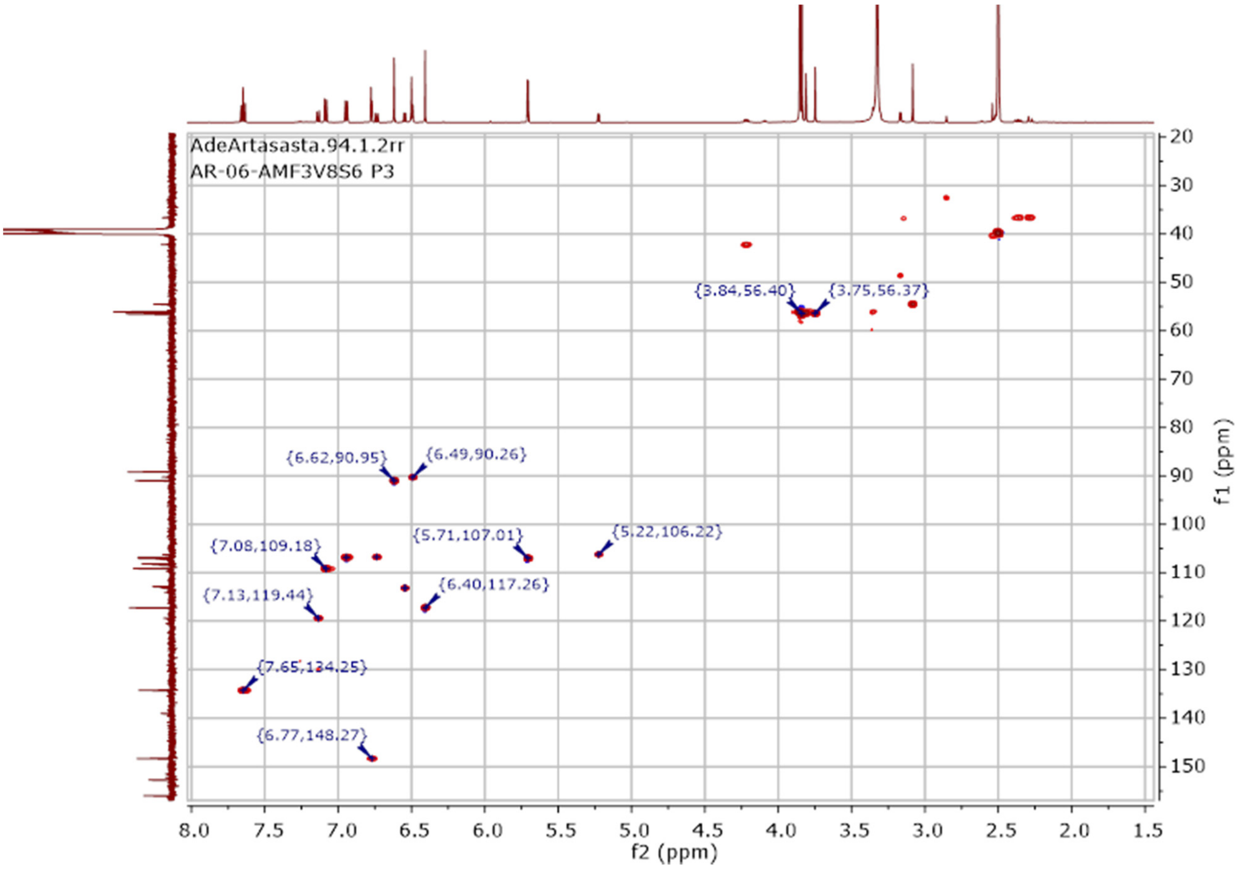

Figure S5. HSQC spectrum of oxisterigmatocystin J.

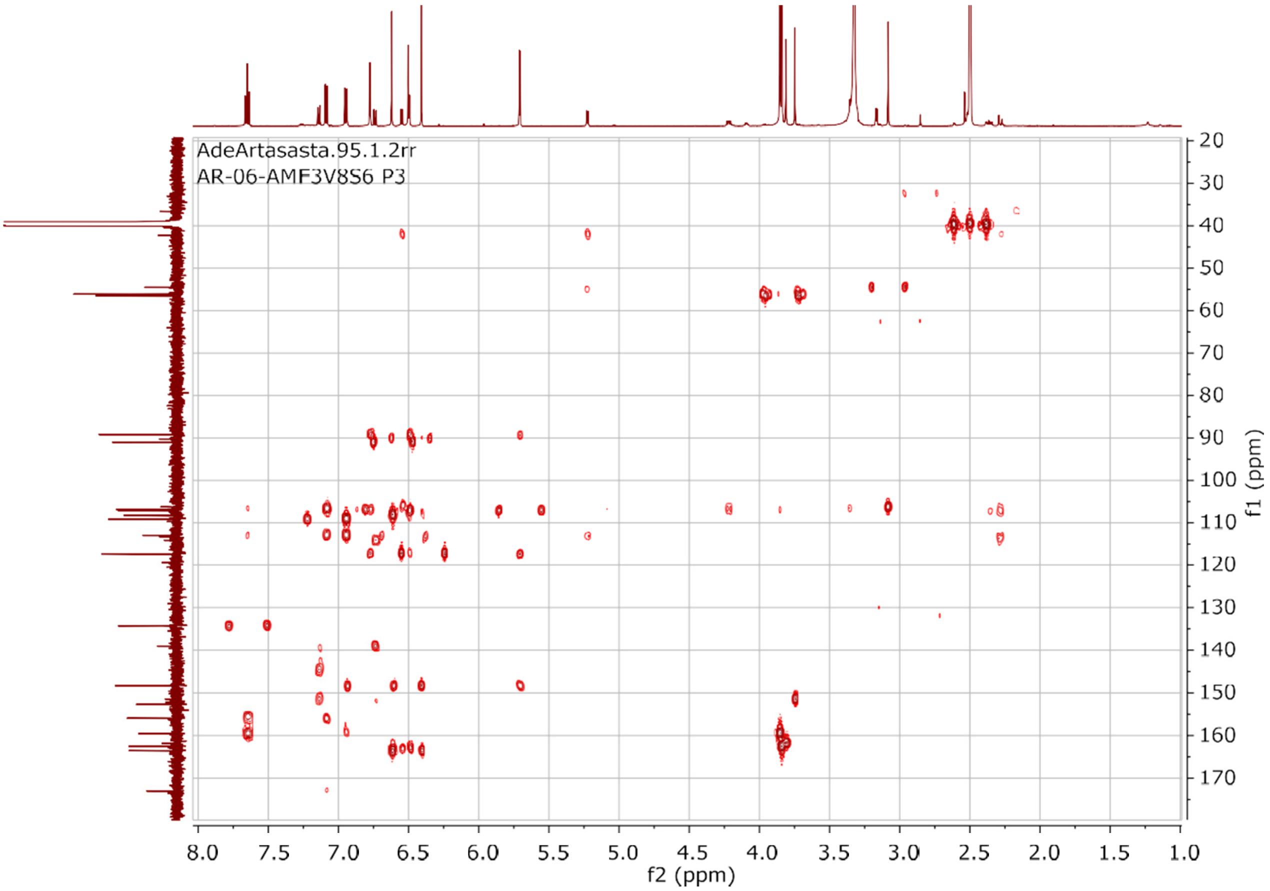

Figure S6. HMBC spectrum of oxisterigmatocystin J.

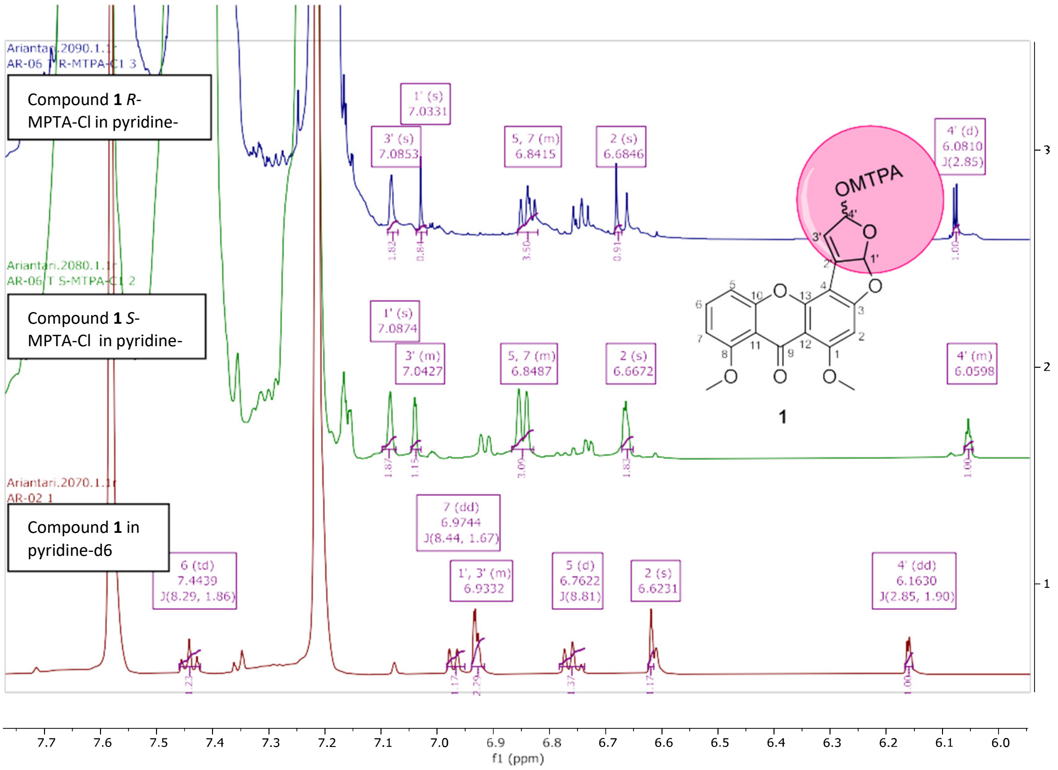

Protons	H-1′	H-3′
(*S*)	7.0874	7.0427
(*R*)	7.0331	7.0853
$^{S-R}$ = (*S*) – (*R*)	+0.0543	-0.0426

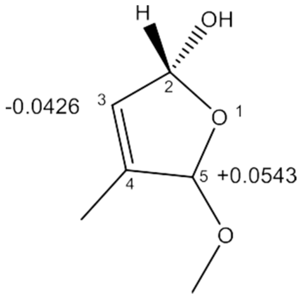

(2*R*)-5-methoxy-4-methyl-2,5-dihydrofuran-2-ol

Figure S7. Mosher results for oxisterigmatocystin J (**1**), spectral assignments were confirmed by ^{1}H-^{1}H COSY.

Oxisterigmatocystin K (2)

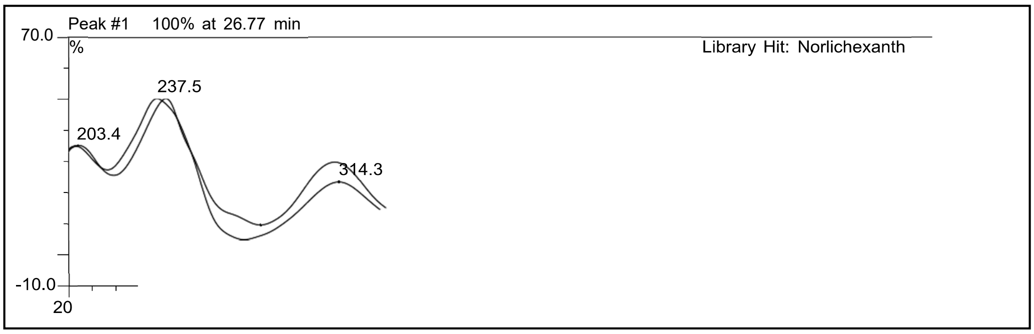

Figure S8. UV spectrum of oxisterigmatocystin K.

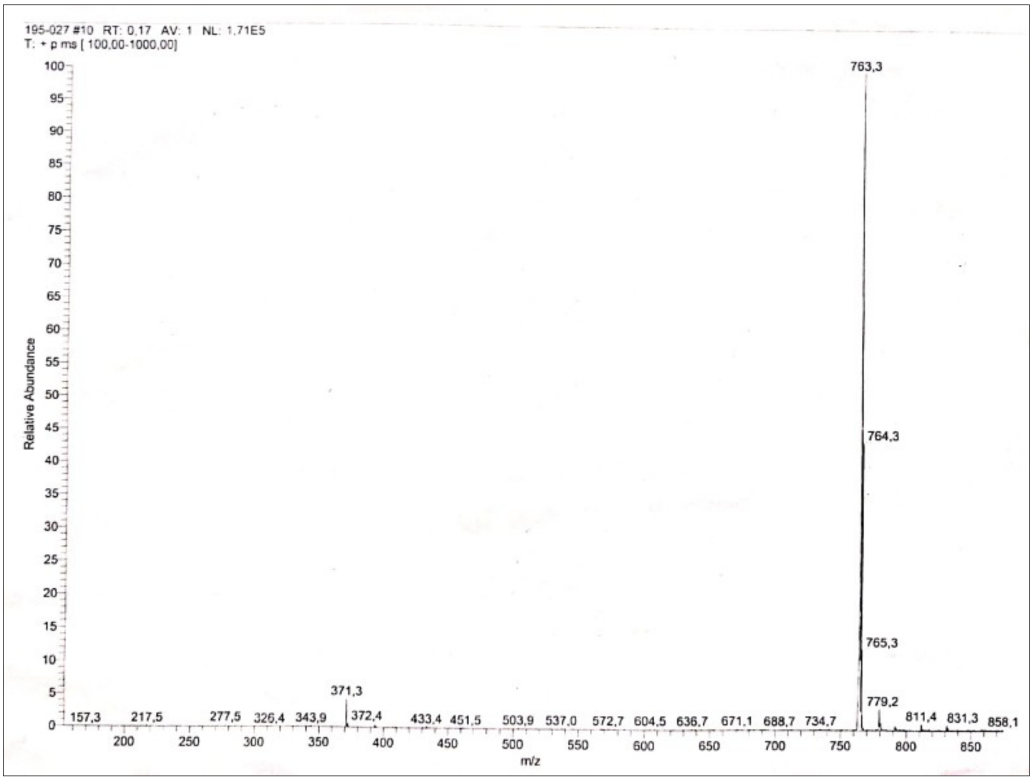

Figure S9. Mass spectrum of oxisterigmatocystin K with m/z ion of $[M + H]^+$ 371.3.

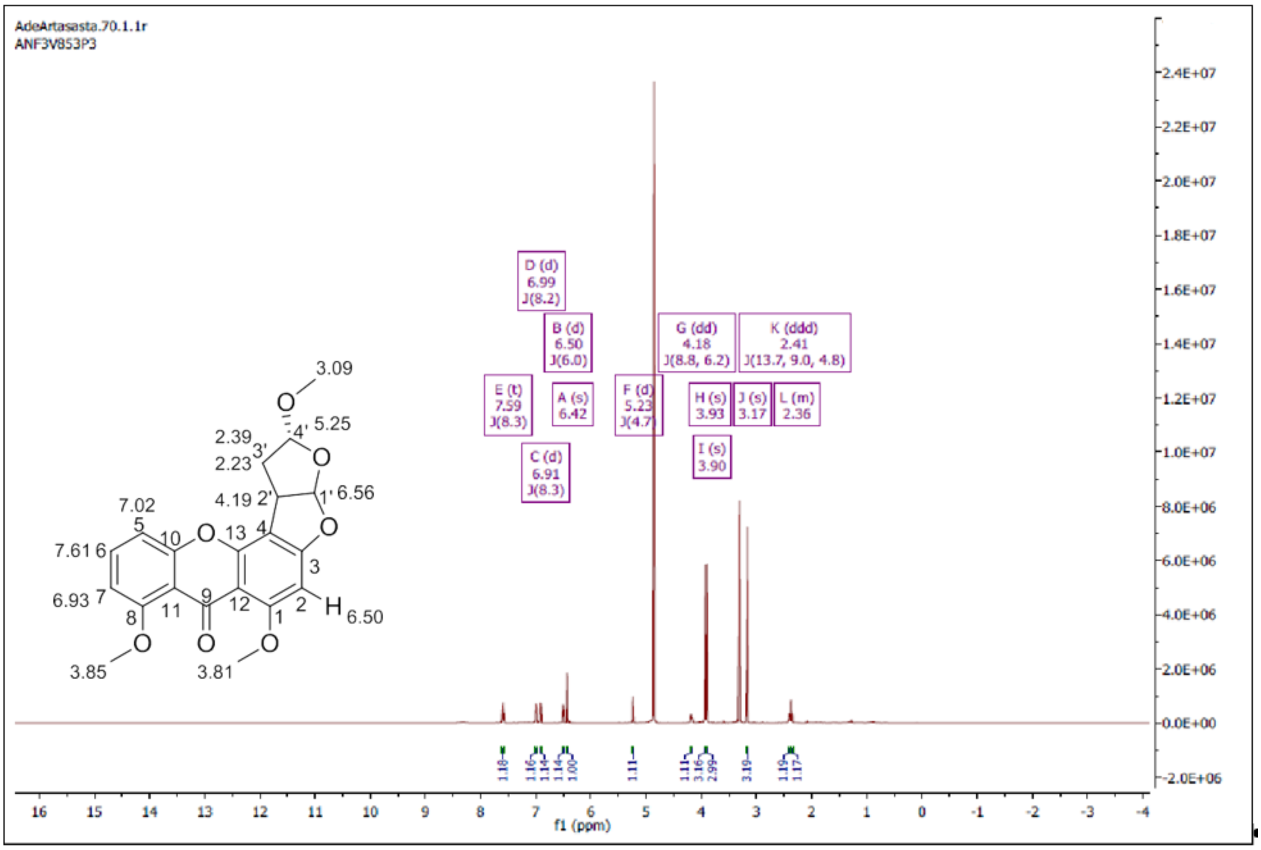

Figure S10. ^{1}H-NMR spectrum of oxisterigmatocystin K.

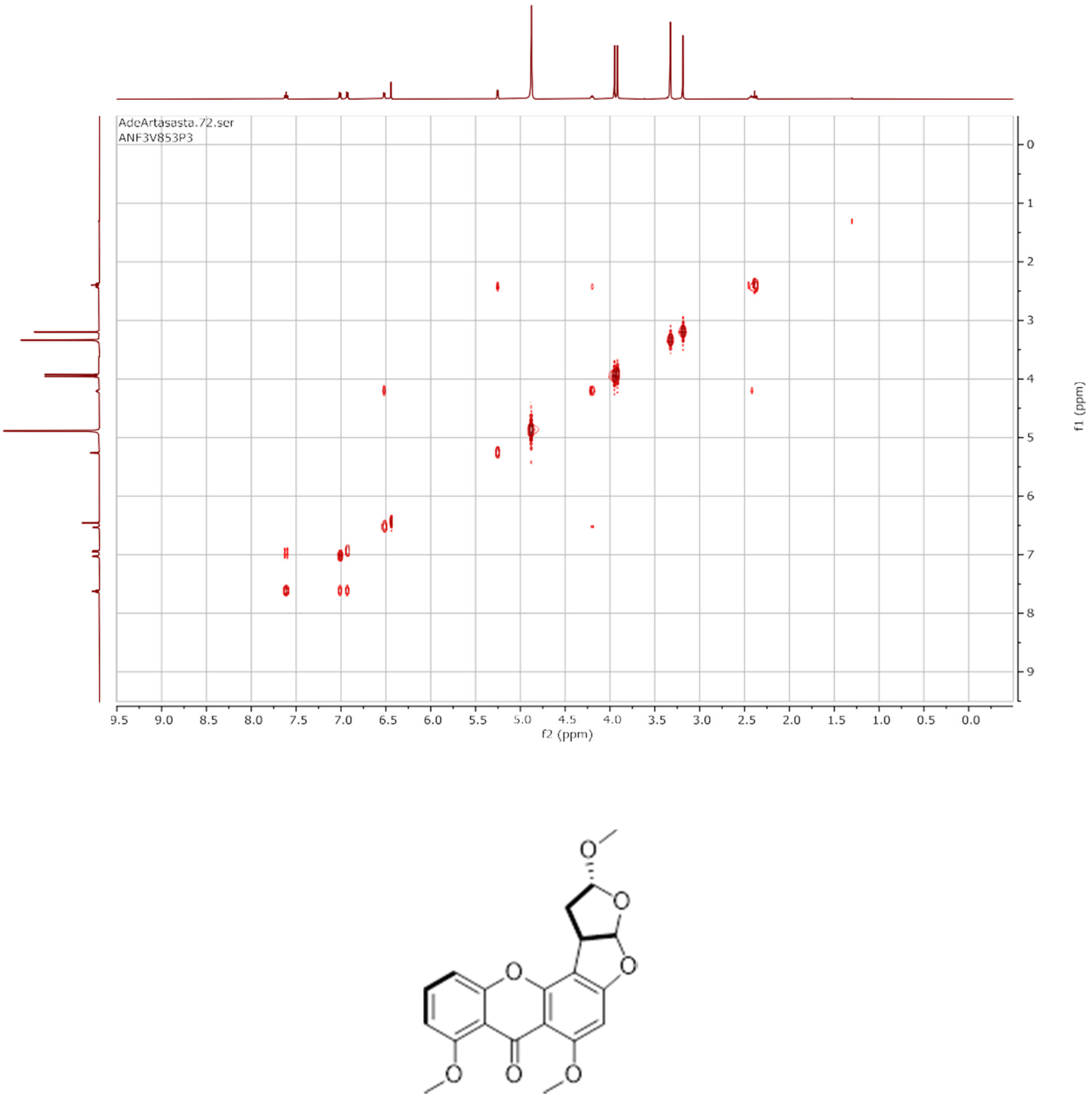

Figure S11. COSY spectrum of oxisterigmatocystin K; proton correlations were shown inblack.

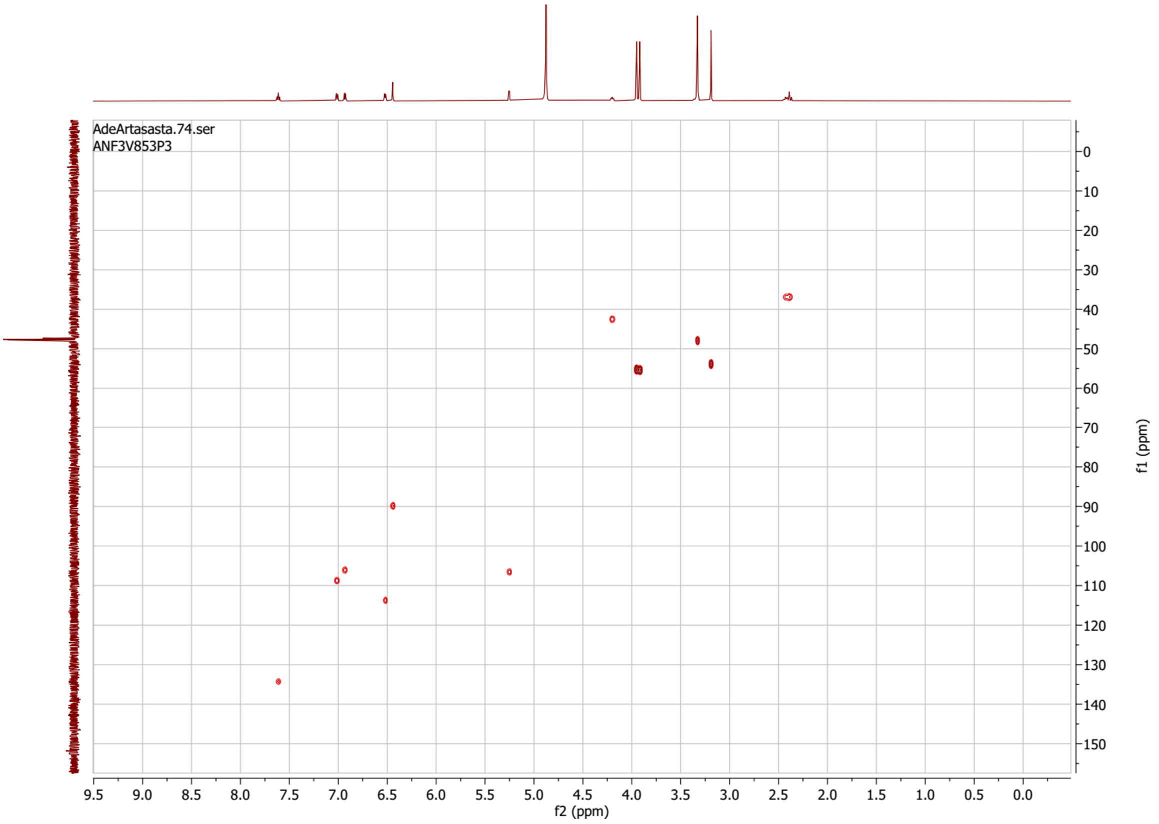

Figure S12. HSQC spectrum of oxisterigmatocystin K.

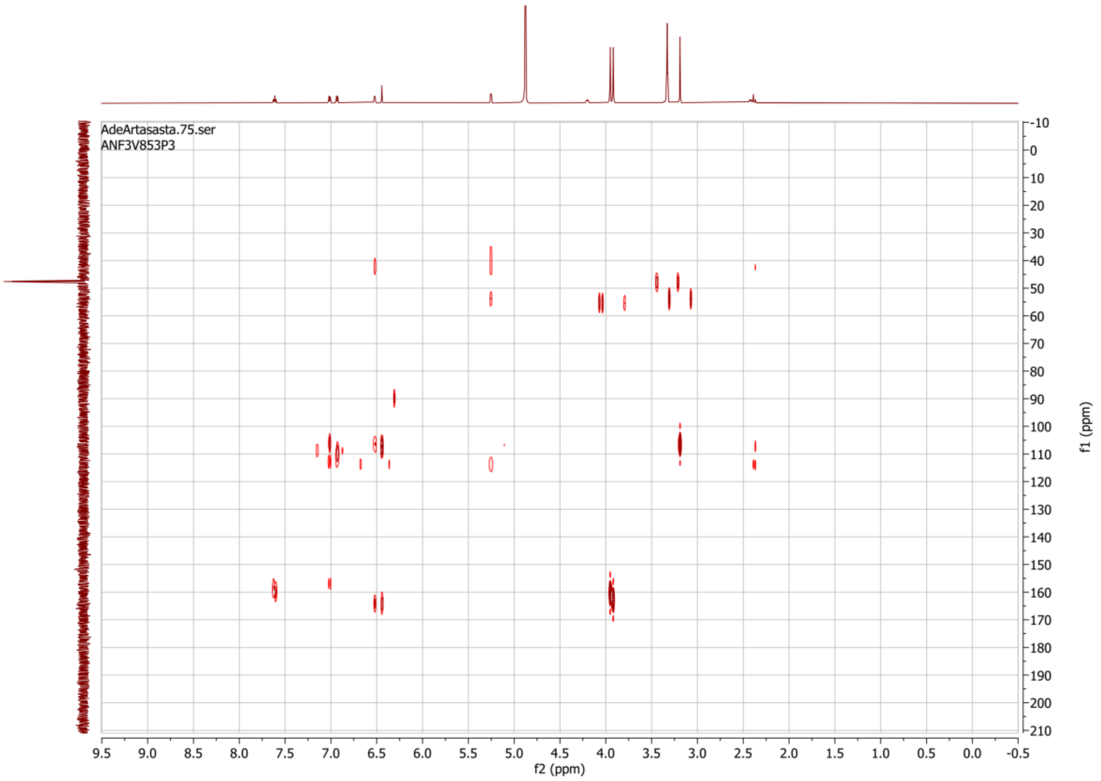

Figure S13. HMBC spectrum of oxisterigmatocystin K.

Oxisterigmatocystin L (3)

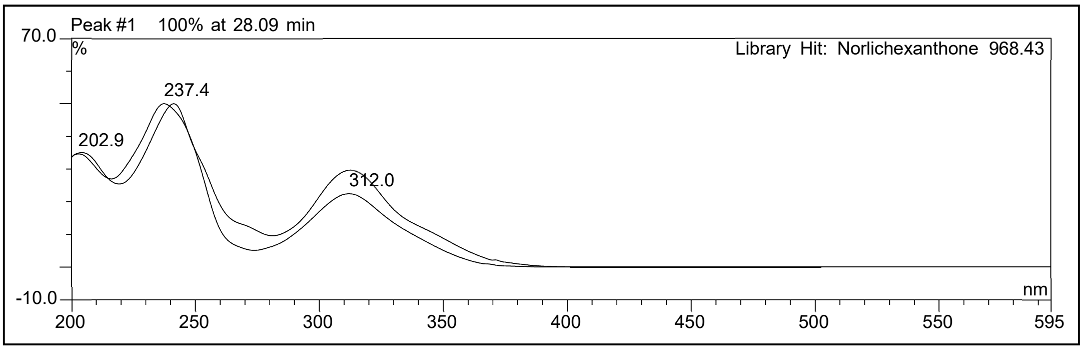

Figure S14. UV spektrum of oxisterigmatocystin L.

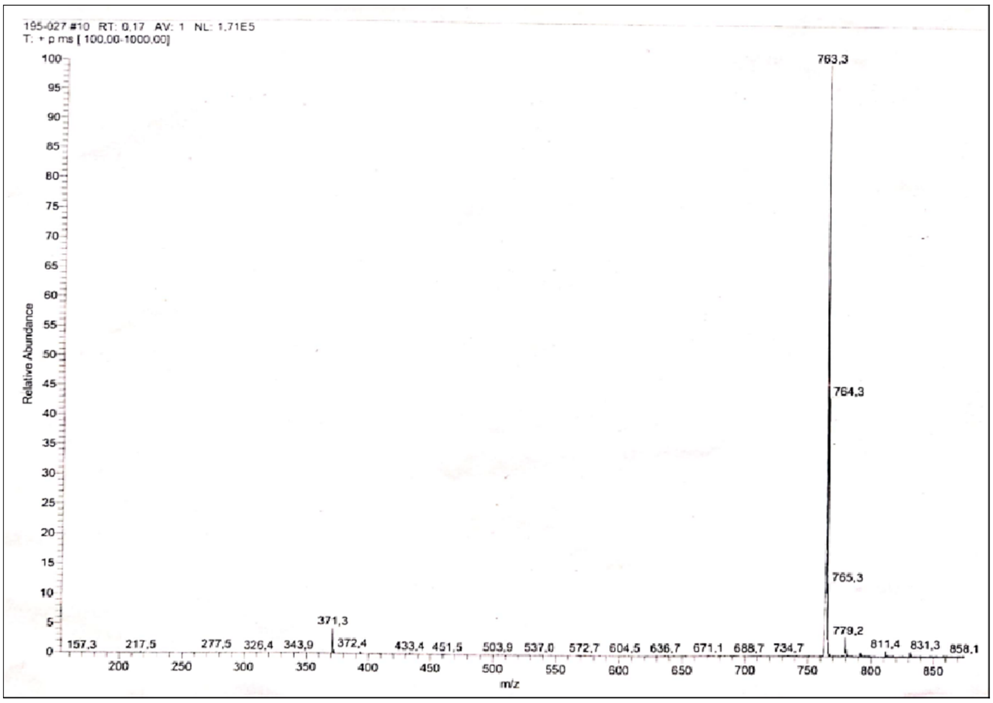

Figure S15. Mass spektrum of oxisterigmatocystin L with *m*/*z* ion of $[M + H]^+$ 371.3.

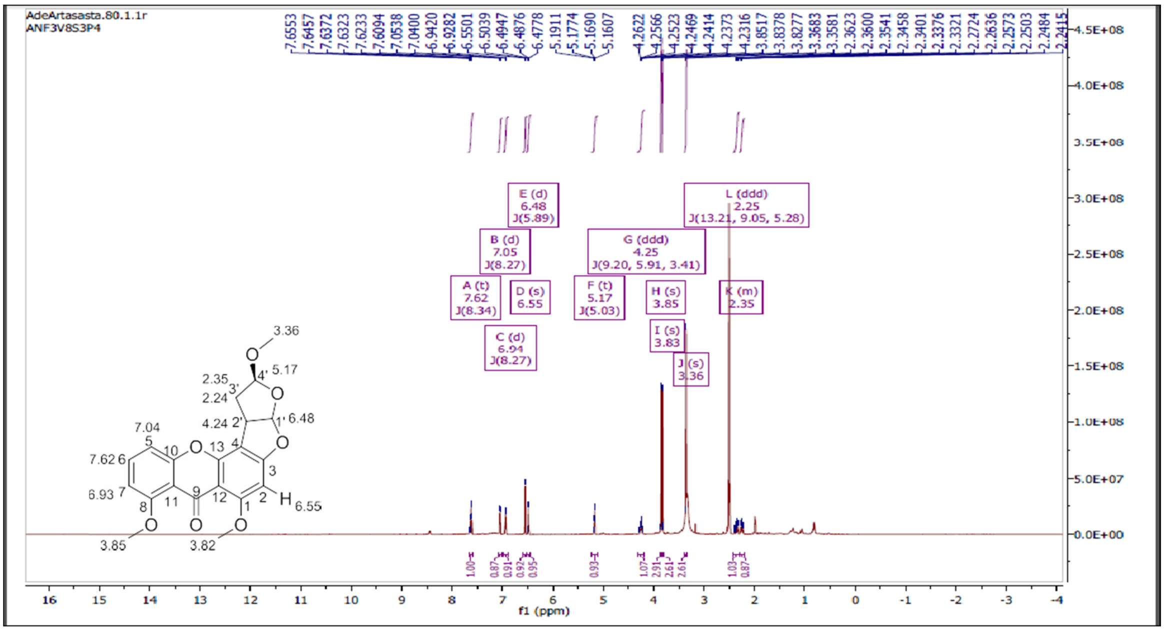

Figure S16. ^{1}H-NMR spectrum of oxisterigmatocystin.

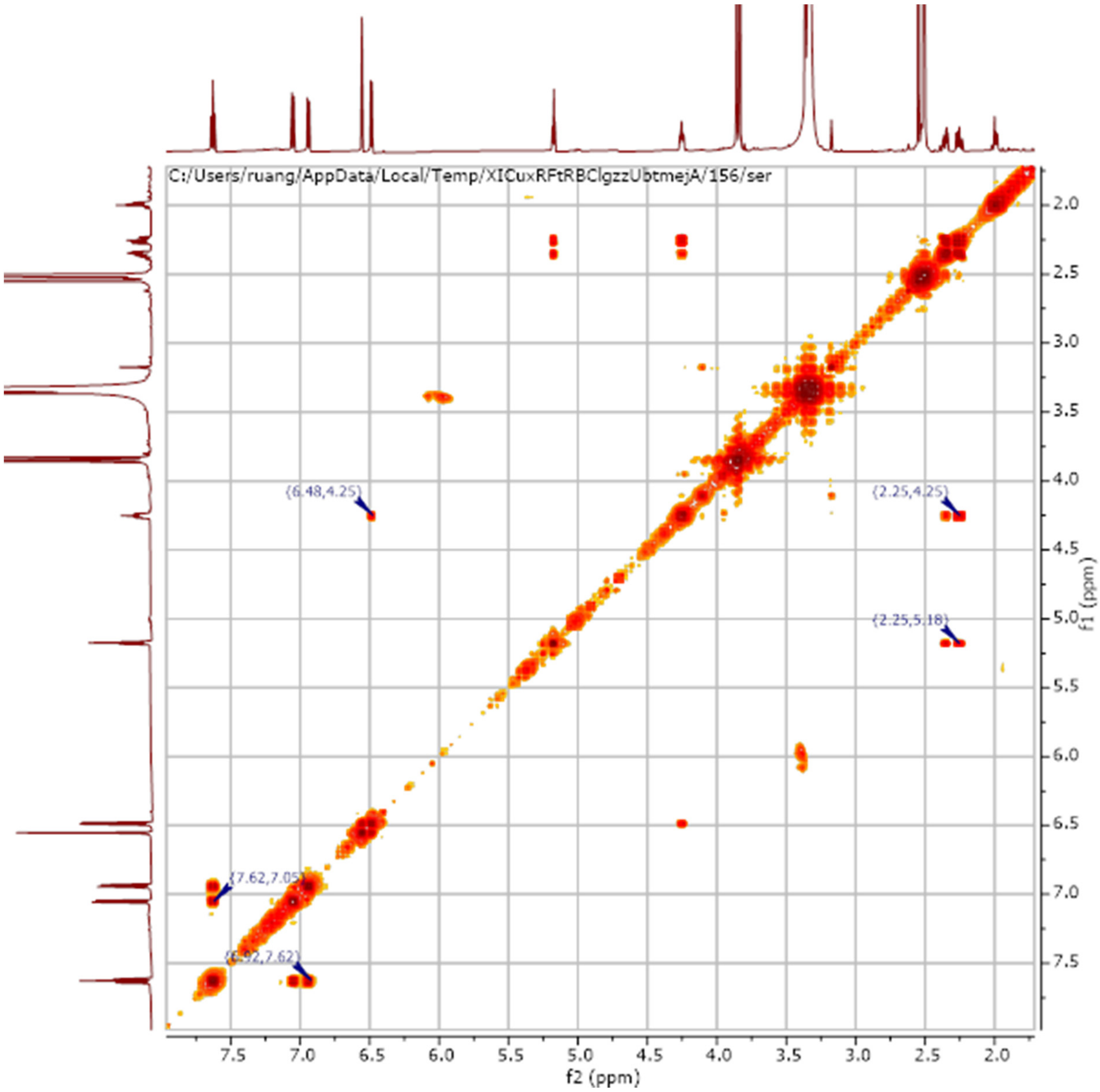

Figure S17. COSY spectrum of oxisterigmatocystin L.

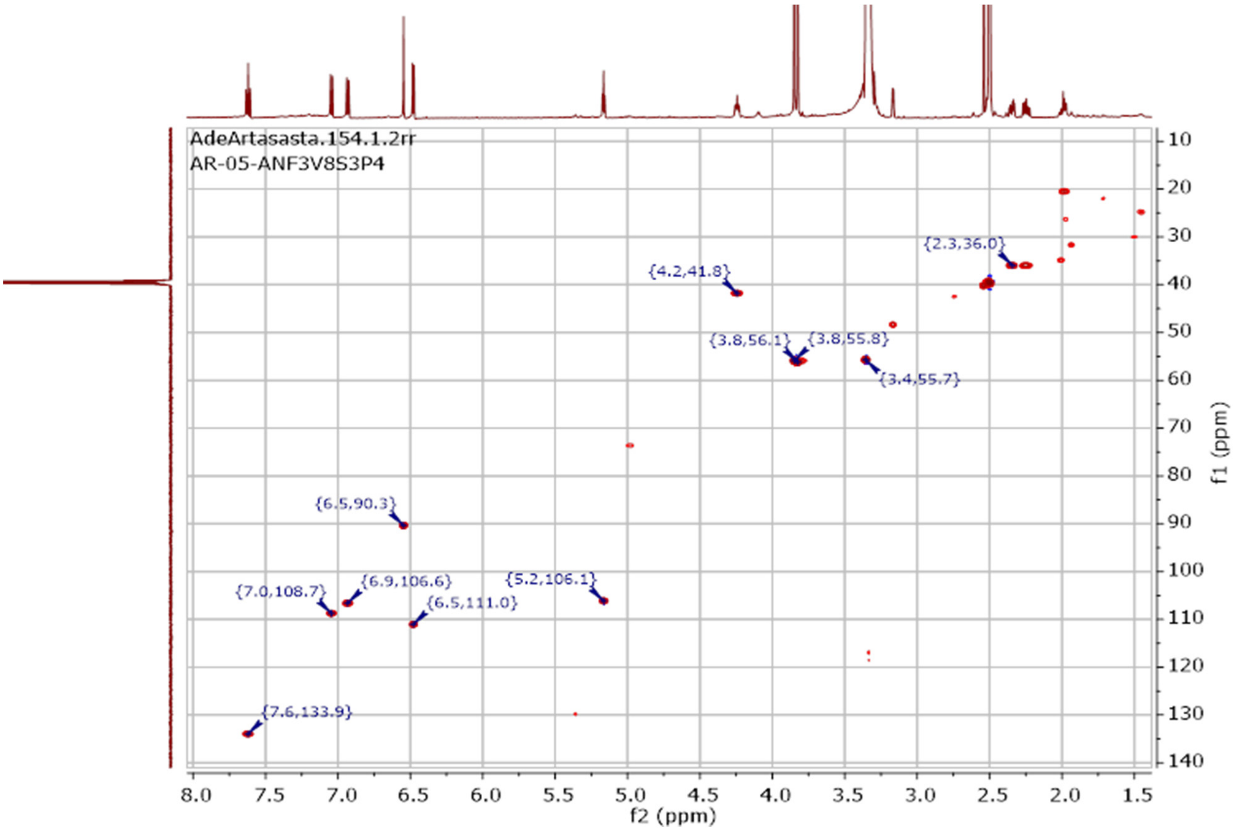

Figure S18. HSQC spectrum of oxisterigmatocystin.

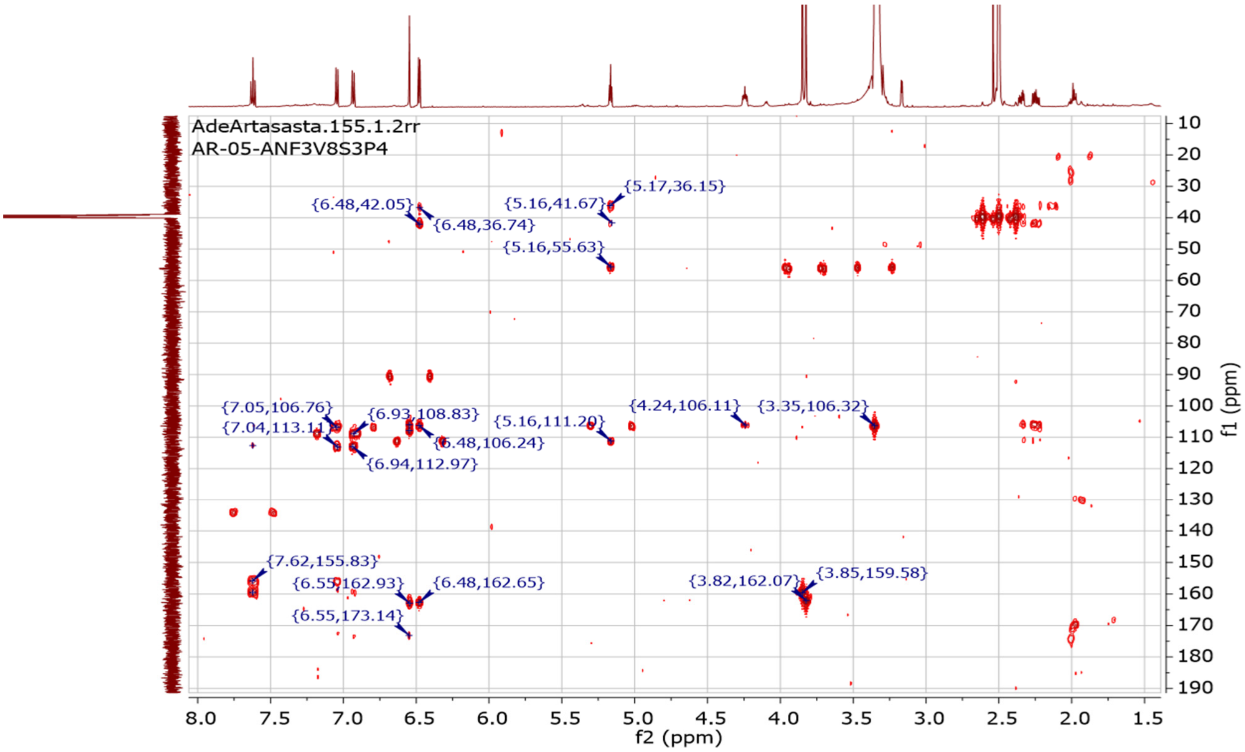

Figure S19. HMBC spectrum of oxisterigmatocystin L.

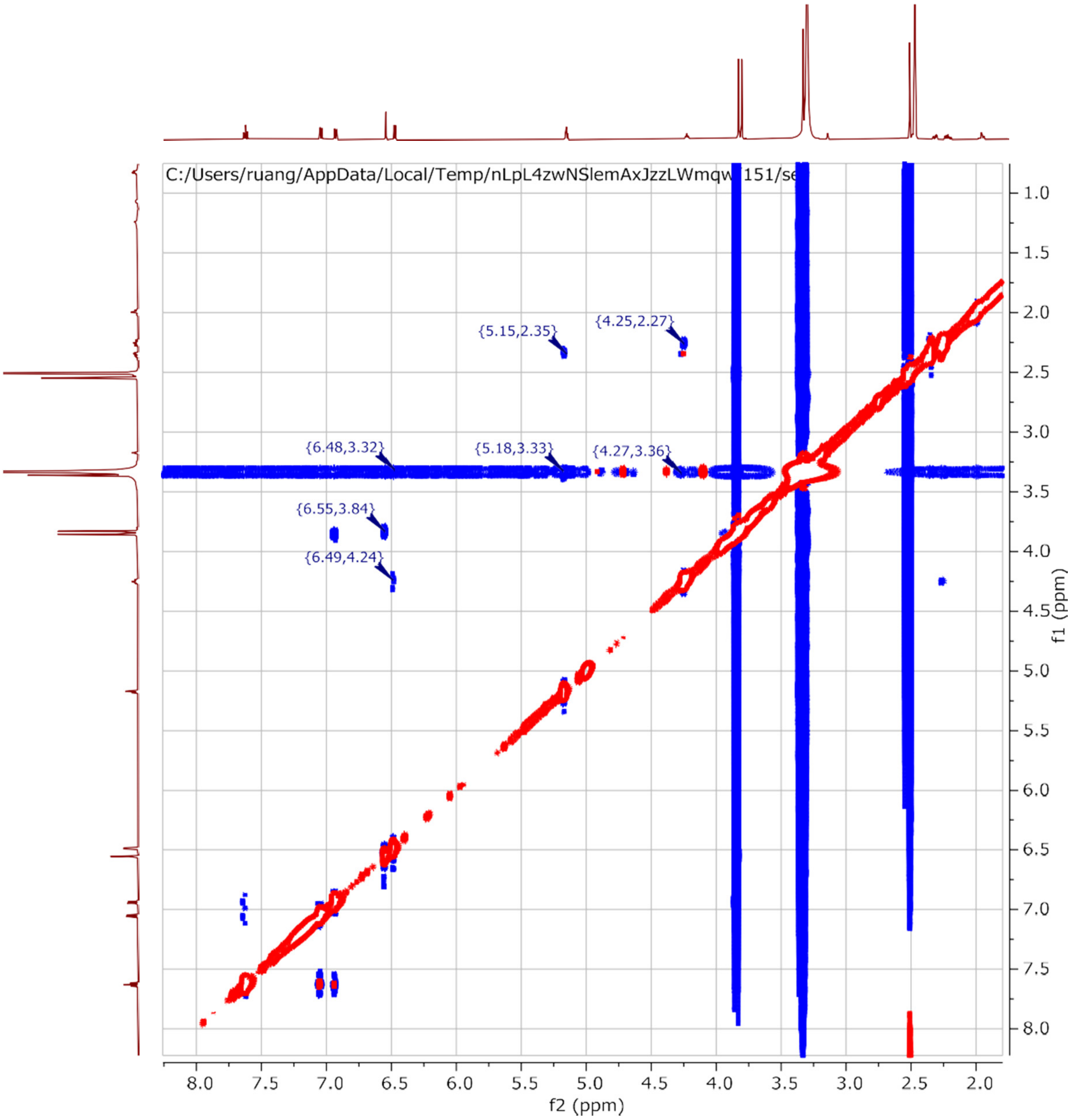

Figure S20. NOESY spectrum of oxisterigmatocystin L.

Aspergillicin A (4)

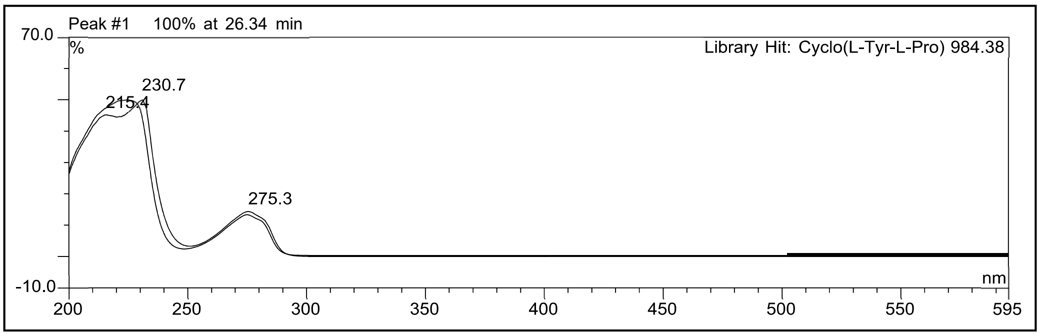

Figure S21. UV spectrum of aspergillicin A.

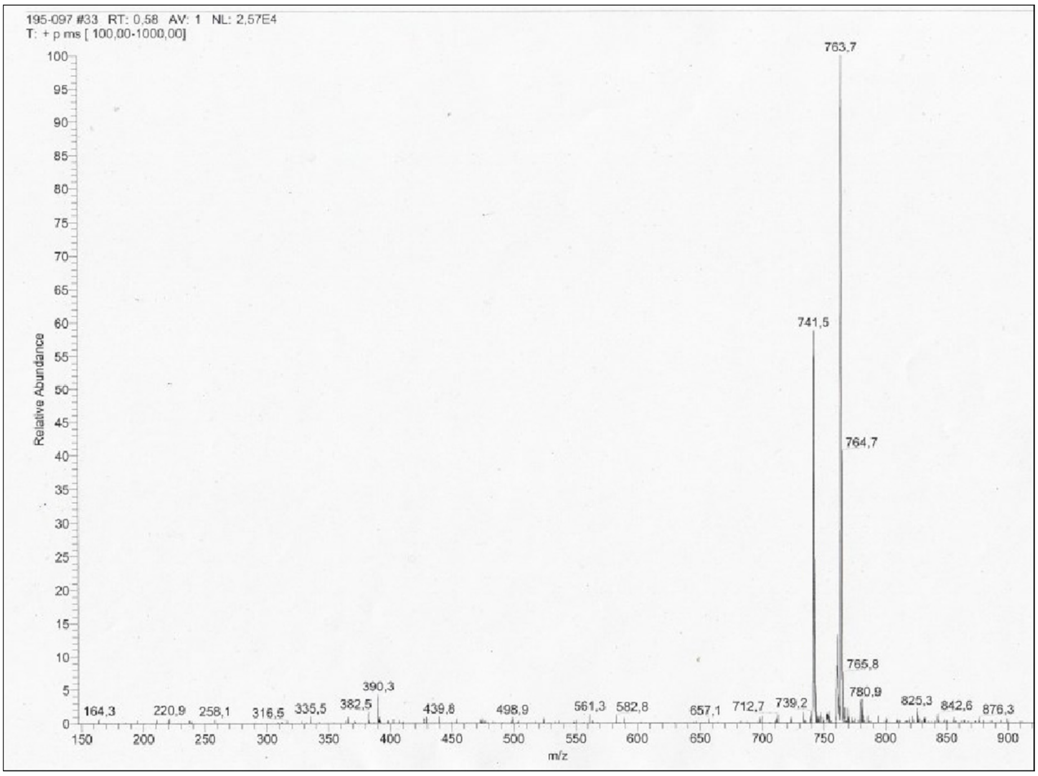

Figure S22. Mass spectrum of aspergillicin A with *m/z* ion of $[M + Na]^+$ 763.7.

Phylogenetic Tree of *A. nomius* NC06

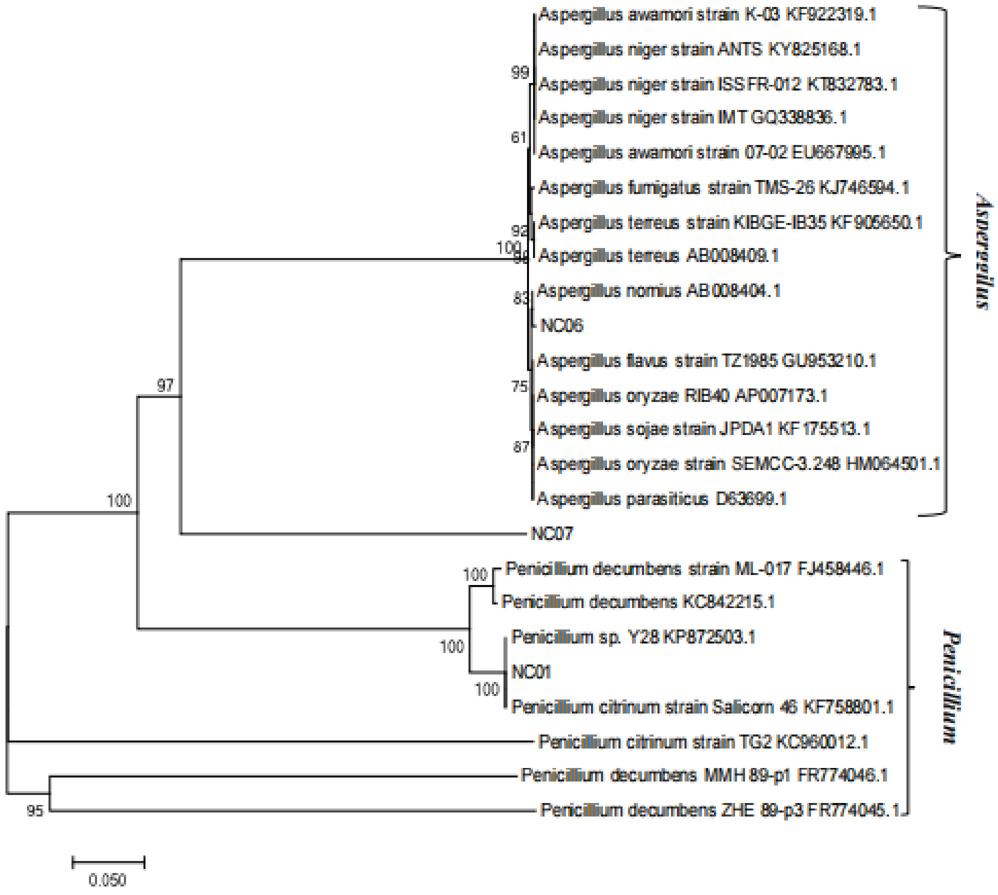

Figure S23. The phylogenetic tree by using neighbors-joining method of 18S Rrna.

The sequenced DNA of this fungus has been published on NCBI with accession number MN242781.1 (https://www.ncbi.nlm.nih.gov/nuccore/MN242781).
